# Twinning Behavior, Microstructure Evolution and Mechanical Property of Random-Orientated ZK60 Mg Alloy Compressed at Room Temperature

**DOI:** 10.3390/ma16031163

**Published:** 2023-01-30

**Authors:** Chengyu Zhang, Di Wu, Yanda He, Wenyu Pan, Jianqiu Wang, Enhou Han

**Affiliations:** 1 CAS Key Laboratory of Nuclear Materials and Safety Assessment, Institute of Metal Research, Chinese Academy of Sciences, 62 Wencui Road, Shenyang 110016, China; 2 School of Materials Science and Engineering, University of Science and Technology of China, 72 Wenhua Road, Shenyang 110016, China; 3 School of Nano Science and Technology, University of Science and Technology of China, 166 Renai Road, Suzhou 215127, China; 4 School of Materials Science and Engineering, Shenyang University of Chemical Technology, 11th Street, Shenyang Economic and Technological Development, Shenyang 110142, China; 5 Institute of Corrosion Science and Technology, 136 Kaiyuan Avenue, Guangzhou 510530, China

**Keywords:** Mg alloy, twinning, random orientation, compression, room temperature

## Abstract

In this study, the uniaxial compression of random orientation ZK60 Mg alloy to different strains was performed at room temperature. The microstructure evolution was characterized mainly using electron backscattered diffraction (EBSD), and the mechanical property was evaluated by the Vickers hardness test. During compression, extension twins nucleated, grew, and engulfed the grain. Twins form a texture with the c-axis parallel to the compression direction. With the massive nucleation and expansion of extension twins during compression, the twin boundary (TB) brought the grain refinement, and the twin boundary-dislocation interaction significantly increased the strain hardening rate of ZK60 Mg alloy, both leading to its significantly increasement of the hardness.

## 1. Introduction

Mg alloy is the lightest structural metal, which can play an important role in energy saving and emission reduction. In industry, over 80% of Mg components are made by high-pressure die casting [1], but casting brings defects such as porosity and shrinkage cavity [2,3]; these defects reduce the consistency of mechanical properties and limit the widespread use of Mg alloys. In contrast, the Mg alloy processed by rolling, extrusion, and forging can reduce casting defects, refine the structure and improve mechanical properties [4,5,6,7]. Some wrought Mg alloys made by rotary swaging have a strength of over 700 MPa [5,8], which shows the ample potential of Mg alloy for application.

As a result, studying the microstructure evolution during the plastic forming is critical. In the mechanical deformation process, dislocation slipping and twinning are the main deformation methods. For dislocation slipping, non-basal slip is difficult to activate at room temperature. This phenomenon makes the Mg alloy prone to cracking during cold working [9]. Then, thermal processing of magnesium alloys was developed to promote the activation of non-basal slip by increasing the processing temperature. Although the activation of non-basal slip can be promoted by heating, Mg alloy has a high thermal conductivity, which is significantly higher than aluminum alloy and steel [10,11,12]. This physical property causes the temperature of Mg alloy dropping rapidly during processing, which results in a narrow processing window for Mg alloy, making it difficult to ensure that the Mg alloy ingots are deformed within the ideal temperature range. When the temperature is below the processing window, the large strain will lead to cracking and strength inhomogeneity of the material [13]. In contrast, the CRSS of twinning is almost independent of temperature, so the control of Mg alloy microstructure by twinning at room temperature becomes the preferred choice.

Twinning is the crystallographic shear process in grain which can change the grain orientation for a certain angle. Twin boundary also can divide the matrix and brings grain refinement. At the same time, twin boundaries as two-dimensional lattice defects can be recrystallization nucleation sites, which facilitate recrystallization [14]. It is evident that twinning can be used to improve the mechanical properties of Mg alloys. As mentioned above, twinning can effectively regulate the microstructure and improves the mechanical properties of Mg alloys. We can promote twinning by reducing the temperature since the CRSS of twinning is less affected by temperature. The CRSS of {10-12} extension twinning is close to the basal slip in Mg alloy [15], which means the {10-12} twinning can be activated easily. This means that it is feasible to regulate the microstructure of Mg alloys by twinning.

In previous studies, it has been found that there are many factors affecting the twinning behavior of Mg alloys, such as temperature [16], alloy composition [17,18,19], strain [20], etc. Therefore, we need a lot of systematic research to regulate the twinning behavior in Mg alloys [21]. Although some studies have been carried out on twinning in Mg alloys, the twinning behavior is not fully investigated at present, and twinning behavior is complex and needs to be studied in more depth. For example, rare earth elements can significantly inhibit twinning and producing the unusual {11-21} twins [22,23]. In addition, the adding of reinforcing phase in the magnesium alloy can effectively promote the nucleation of twin variants [24].

Strain also significantly affects the evolution of twinning behavior of Mg alloys during processing, which will have a significant effect on the final properties of Mg alloys. Many twinning behavior of single-component Mg alloys has been studied, but it is not detailed and thorough enough [25,26]. In order to make full use of the regulation of Mg alloy twinning on the microstructure, more studies are needed on the twinning behavior of Mg alloys from small strains to failure. In addition, the current studies are based on hot-worked, strong-textured Mg alloys, and there is a lack of a specific study of a particular Mg alloy, especially in different strains.

ZK60 Mg alloy is the typical high-strength wrought Mg alloy, and advanced processing has been widely used on it [27,28]. In this article, the cast ZK60 Mg alloy with random orientation was selected, and deformed at room temperature. EBSD was mainly used to analyze its twinning behavior, and related microstructure evolution, texture and mechanical properties were also investigated.

## 2. Materials and Methods

### 2.1. Materials Preparation

The material used in this research is a commercial cast ZK60 (Mg-Zn-Zr) alloy in homogenized state; Table 1 presents the chemical composition of this alloy. In this study, the cylinder compression sample was used, with dimensions Φ10 × 15 mm, machined by wire electrical discharge machining.

### 2.2. Microstructure Characterization

The phase composition of the specimen was identified by X-ray diffraction analysis (XRD) (Rigaku D/Max-2500 PC, Tokyo, Japan) with a scan speed of 2°/min. The metallography was observed by optical microscope (OM) (Zeiss Axio Observer Z1, Jena, Germany). The microstructure and the element distribution were observed and analyzed by environmental scanning electron microscope (ESEM) (FEI XL30 FEG, Eindhoven, The Netherland) equipped with energy dispersive spectrometer (EDS) (EDAX, Oxfordshire, UK). Transmission electron microscope (TEM) (JEOL J2100F, Tokyo, Japan) was also used to analyze the detailed microstructure of the ZK60 Mg alloy.

### 2.3. Electron Backscatter Diffraction Analyzation

The electron backscatter diffraction (EBSD) specimens were firstly grinded with abrasive paper from 200# to 5000#, and electrochemical polishing was used at 15 V with the electrolyte (90% ethanol and 10% perchloric acid) for the 30 s. The EBSD analysis was conducted by ESEM (Thermo Scientific Quattro S, Waltham, MA, USA) equipped with EBSD detector (EDAX, DigiView, Mahwah, MA, USA). A 20 KeV electron beam was selected with a spot size of 6.5, and the step size was 2.5 μm. The EBSD data was analyzed by TSL OIM software (Version 8.62).

### 2.4. Room Temperature Compression

**Figure 1:**
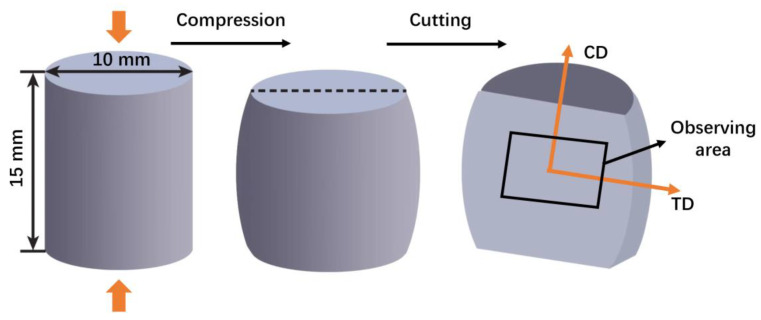
The compression scheme and EBSD sample coordinate system.

The specimens were compressed at room temperature with a strain rate of 0.001 s^−1^. The compression experiment works on the universal testing machine (Shimadzu, AG-X 50 kN, Kyoto, Japan). A schematic of the experimental approach and sampling observation is illustrated in Figure 1, and the reference directions are defined as CD (compression direction) and TD (transverse detraction).

### 2.5. Hardness Test

The Vickers hardness test used the MHVD-1000 AP microhardness tester (Shanghai Optics And Dine Mechanics Institution, Chinese Academy of Sciences, Shanghai, China) with an applied load of 500 g and a holding time of 15 s. For experimental confidence, every specimen was tested eight times at the core area.

## 3. Results

### 3.1. Microstructure Characterization of Initial ZK60 Alloy

**Figure 2:**
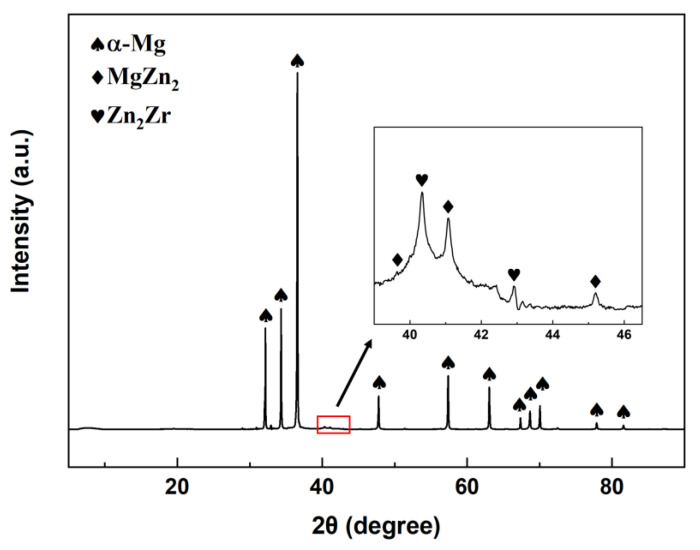
XRD pattern of cast ZK60 Mg alloy.

**Figure 3:**
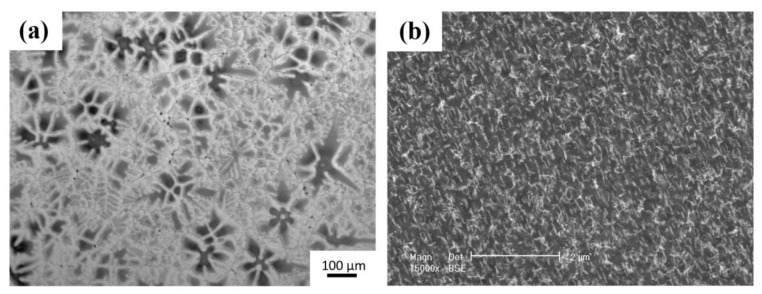
(**a**) Metallograph and (**b**) SEM image of cast ZK60 Mg alloy.

Figure 2 shows the XRD pattern of the initial ZK60 alloy. The cast ZK60 alloy is mainly composed α-Mg, and a small amount of MgZn_2_ and Zn_2_Zr phase. Figure 3 shows the OM and SEM images of the as-homogenized alloy. The initial microstructure consists polygonal grains, and most grain size was over 200 μm. The petaloid patches were observed in the interior of the matrix, and some bulk-shaped phases were distributed randomly at the grain boundary. Figure 3b is the high magnification image of petaloid patches, and a high-density rod precipitated phase was observed.

**Figure 4:**
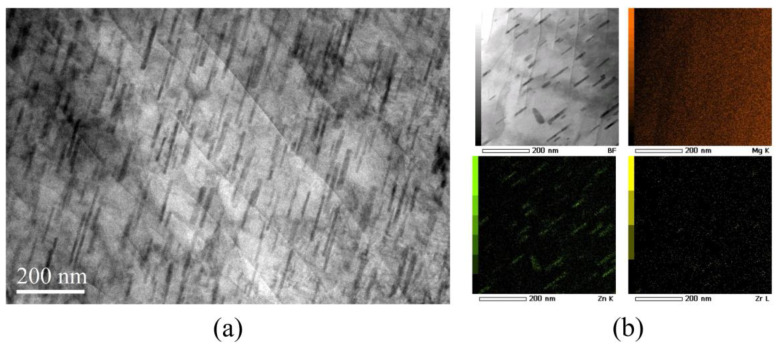
(**a**) TEM image and (**b**) STEM/EDS mapping of cast ZK60 Mg alloy.

**Figure 5:**
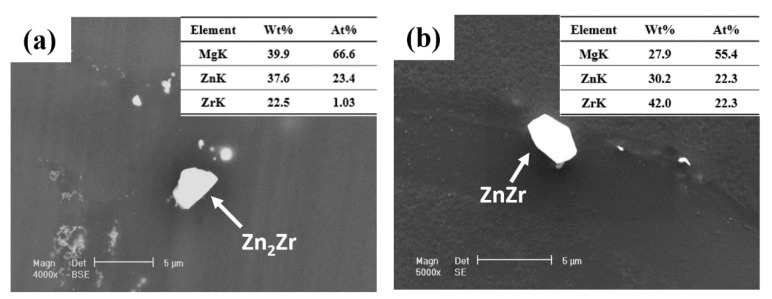
(**a**,**b**) the SEM image of particle phase at the grain boundary, and corresponding spot spectrum EDS results of bulk-shaped phases at grain boundary.

Figure 4a is the TEM image of the initial ZK60 Mg alloy. The rod precipitated phase has the preferred orientation, which has been confirmed is parallel with the c-axis of the grain [29,30]. Figure 4b shows the bright filed STEM image and EDS mapping of dense distribution of rod phase. From the EDS mapping, we can see the Zn element congregate at the rod phase. Combined with XRD results, the rod-like precipitated phase can be identified as MgZn_2_. Figure 5 shows the SEM-EDS results, and bulk-shaped phases at the grain boundary were Zn_2_Zr and ZnZr, which was also identified in the XRD pattern.

### 3.2. The Compression Curves

**Figure 6:**
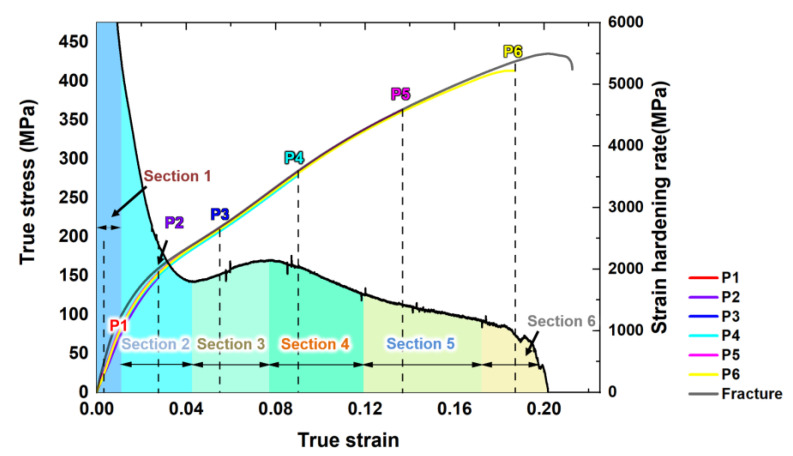
The true stress-strain curve and strain hardening rate-true strain curve.

Figure 6 shows the true stress-strain curve and strain hardening rate-true strain curve. The concave down of the true stress-strain curve and the uplift of strain hardening rate-true strain curve all manifest the main deformation mechanism of ZK60 Mg alloy is {10-12} extension twinning [20,31,32]. Table 2 shows the strain of each point in Figure 6. For points P1 and P2, which are points with small strains, they are mainly used to observe the strain range in which twinning occurs and whether twinning occurs at small strains. For point P3, this is the point where the strain hardening rate appears to rise significantly near the middle of the range taken. Point P4 is located in the area where the strain hardening rate falls rapidly again after the highest point, and point P5 in the area where the decreasing trend of strain hardening rate slows down. For the region where P6 is located, there is a significant decrease in strain hardening rate and fracture may have occurred in this region.

### 3.3. Microstructure Evolution

**Figure 7:**
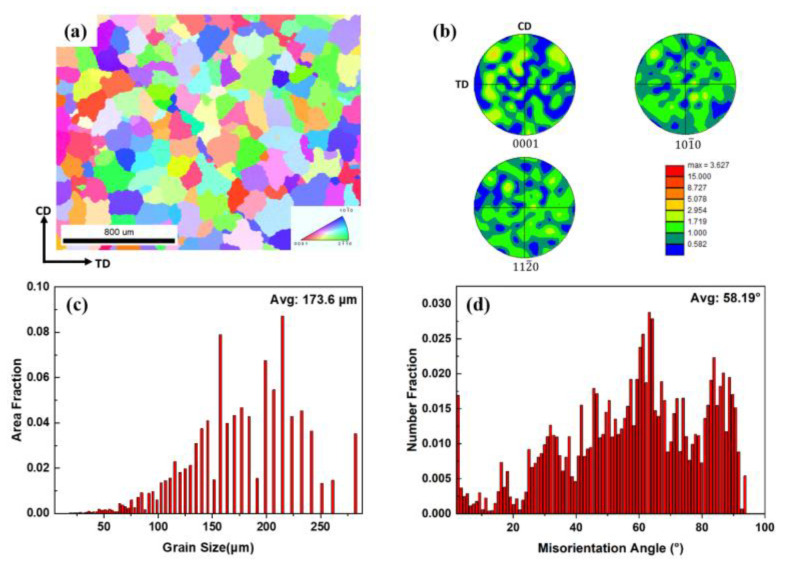
(**a**) The inverse pole figure (IPF) map, (**b**) pole figure, (**c**) grain size, and (**d**) misorientation angle distribution of as-homogenized ZK60 Mg alloy.

From Figure 7, the EBSD results show that the initial alloy has equiaxed grains with curved boundaries, and the pole figure (PF) shows the initial alloy exhibits low texture intensity and multi-peaks, which means the grains have a random orientation. Figure 7c shows that the grain size is mainly distributed from 140 to 240 μm, and the average grain size is 173.6 μm. Because of the noise and some tiny grains. The statistical grain size can have a small gap from the actual grain size. Figure 7d exhibits that the grain boundaries are mainly high-angle grain boundaries (HAGBs).

**Figure 8:**
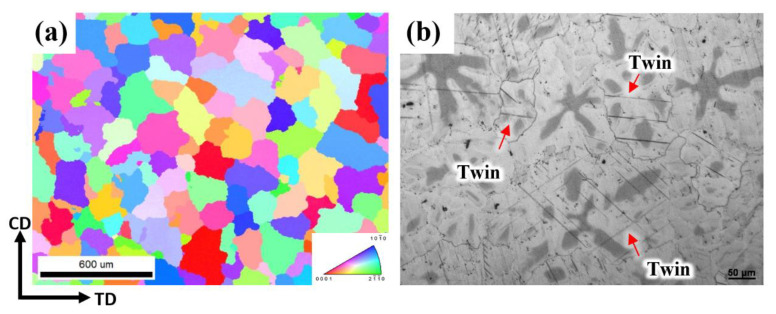
(**a**)The IPF map and (**b**) metallography at P1.

From Figure 8, the IPF map shows no twins nucleated at P1, but in metallography, small twins can be observed. The tiny morphology and the 2.5 μm step size keep the twins from being indexed by EBSD. At P1 the stress and train were 23.8 MPa and 0.25%. In Koike’s work the {10-12} extension twin CRSS of polycrystalline Mg alloys is activated at 2–2.8 MPa [33]. Lu et al. found twin nucleates under the strain of 0.3% [34], and Chen et al. found the twins nucleated at the strain of 0.25% [35]. These previous works confirm that twins can be nucleated at low strain and stress.

**Figure 9:**
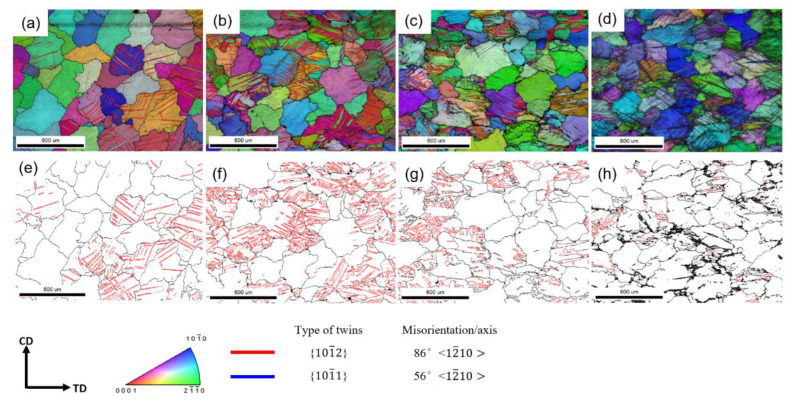
The IPF + IQ + GB map of the specimens at (**a**) P2, (**b**) P3, (**c**) P4, (**d**) P5, and the corresponding GB map of specimens at (**e**) P2, (**f**) P3, (**g**) P4, (**h**) P5.

**Figure 10:**
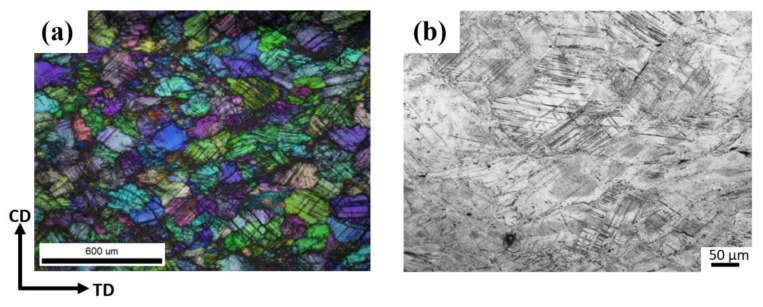
(**a**) The IPF map and (**b**) metallography at P6.

Figure 9 shows the IPF + IQ (image quality) + GB (Grain Boundary) map in the compression process. At P2, twinning can be detected by EBSD. Due to the low CRSS of {10-12} extension twinning, even if the grain orientation is not beneficial to the {10-12} extension twinning, they can still nucleate at P2 [36], and Figure 9a confirms that all twins at P2 are {10-12} extension twins. In P2′s IPF map, we can see that the twins have parallel and cross structure. The cross structure is one kind of work hardening method of Mg alloy [37,38]. Moreover, such cross structure divides the matrix and produces a closed space restricting the dislocation motion [38] and the further expansion of twins [39]. These factors make the cross structure need more force to continue the deformation. At P3, many twins were expanding. In some areas, the twin can merge half the area of the matrix. At P4, some twins expanded to swallow the entire matrix, which means the matrix was covered by the twins, and most of the twin boundaries detected were still {10-12} extension twin boundary. Moreover, some thin twins can be found, and the morphology is the same as {10-11} compression twins. At P5, due to the large strains and stresses, a large number of dislocations interactions formed deformation band in the ZK60 Mg alloy. At P6, the deformation in the specimen is severe, and the index of EBSD is much lower than P1-P5 specimen, and more deformation bands can be observed in Figure 10b.

### 3.4. Orientation Statistics

**Figure 11:**
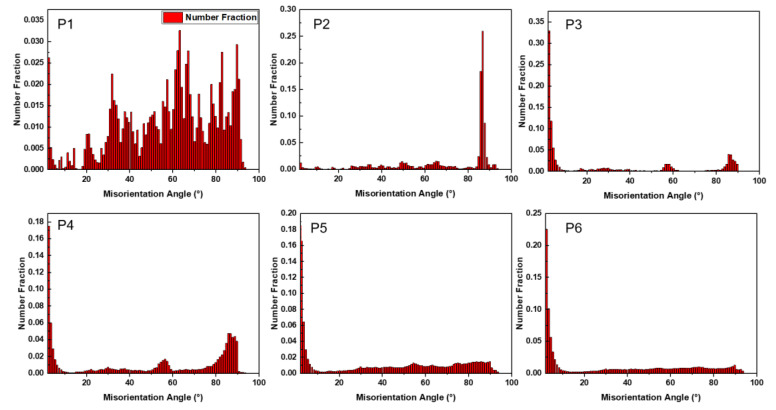
The misorientation angle at P1, P2, P3, P4, P5, and P6.

From Figure 11, P1 shows a random distribution of misorientation angle, which means the microstructure was not explicit changed. At P2, the 86.5° misorientation angle increases rapidly, which means the nucleation and expansion of {10-12} extension twinning. At P3, the fraction of 86.5° decreased slightly, and the low-angle grain boundaries (LAGBs) increased. Compared with Figure 9, the expansion and merging of {10-12} extension twinning was the reason for the decrease of 86.5° peak. At P4, the peak of 86.5° decreased obviously, which means many grains were merged by the twins. Meanwhile, 56.2° misorientation appeared and it means the {10-11} compression twinning nucleate, which is a coincidence with Figure 9g. The LAGBs also increased, obviously. As the strain increased to P5, the misorientation of 86.5° almost disappeared, which means the twinned matrix was all merged by the {10-12} twins. In comparison with that at P5, the fraction of LAGBs at P6 changes less, and the peak of 56.2° becomes less obvious due to the increase in background intensity, and from Figure 10b, we can still see the thin twins.

Table 3 shows that the LAGBs significantly increased than initial. From P1 to P3, the LAGBs increase gradually. From P5 to P6, the LAGBs slightly increased. In the compression process, the density of the dislocation increases with the increase in the strain, which means the entanglement and interaction of the dislocations also increased and formed the LAGBs.

**Figure 12:**
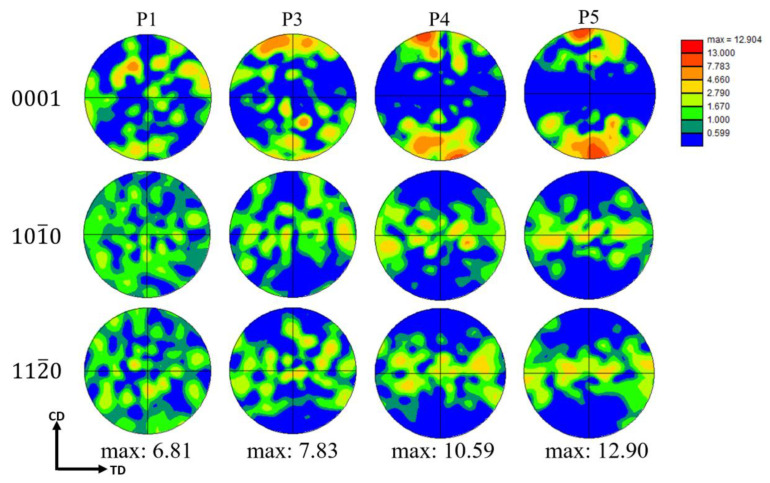
The PF at P1, P3, P4, and P5.

**Figure 13:**
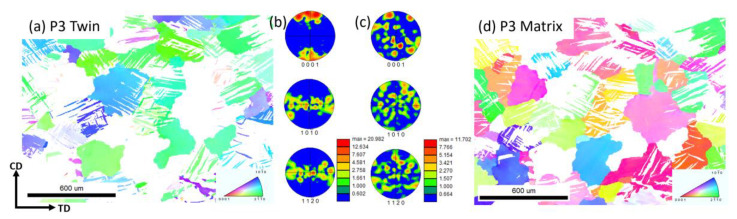
(**a**,**d**) The IPF Map and (**b**,**c**) corresponding PF of the twin and the matrix of P3.

Figure 12 shows the PFs of the ZK60 Mg alloy in the compression process. From the PFs, we can see the basal texture intensity of the specimens increased with the strain. At P4, the basal texture was stable, and P5 had the same texture distribution but higher intensity. The basal texture is the typical texture of Mg alloy where the c-axis of the grain is parallel to the compression direction. In Figure 13, we separated the twin and the matrix at the P3 strain, and it is clear that twinning is the direct reason for the basal texture in the compressed ZK60 Mg alloy, while the matrix maintains a relatively random orientation.

### 3.5. The Evolution of the Schmid Factor

**Figure 14:**
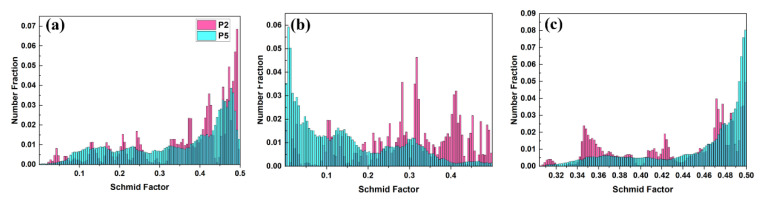
The Schmid factor at P2 and P5: (**a**) the basal slip, (**b**) the prismatic slip, (**c**) pyramidal <**c + a**> slip.

Figure 14 shows the Schmid Factor (SF) at P2 and P5. The basal slip SF has decreased slightly. Although the texture intensity of the Mg alloy increases significantly, the Schmid factor of base slip still has a high level, which means the basal slip remained sufficient to activate. After being compressed to P5, the SF of prismatic slip decreased a lot. Most of the grains have a low SF (<0.2) of prismatic slip, indicating the suppression to prismatic slip. Figure 14c shows the SF of pyramidal <**c + a**> slip becomes higher, which is similar to Gui et al.’s work [40], and the activation of pyramidal <**c + a**> was reasonable with the high stress at P5.

### 3.6. The Interaction between Twin and Participate Phase

**Figure 15:**
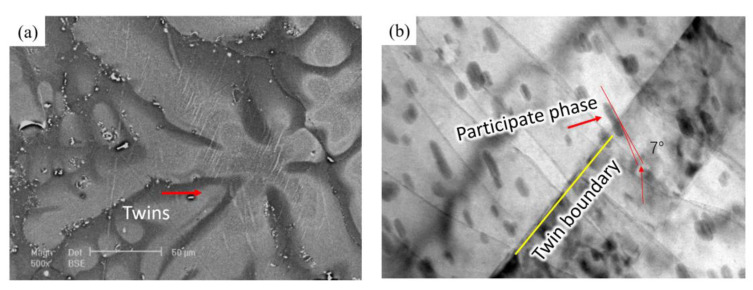
(**a**) The participate phase in the petaloid area, and (**b**) The TEM image of the participated phase at TB.

From Figure 9, we can see that twins did not occur in some grains, the SEM was used to observe the grains. In Figure 15a, the thin twins nucleate with high density was shown. In order to investigate the reason of this phenomenon, TEM was used to observe the relationship between twinning and precipitation phases. From Figure 15b, the TEM image shows that the rod participates was not sheared by twin; it was kinked by a small angle which is the same as Robson’s work [41], and much research shows that the participated phase will inhibit the expansion of twins, which will increase the stress in the matrix. The increase in stress will provide the additional drive force for twinning nucleation [30,41,42], which lead to the thin twins nucleate with high density shown in Figure 15a.

## 4. Discussion

### 4.1. The Relationship between Twinning and Strain Hardening at Room Temperature

From Section 3, we can divide the evolution of microstructure into four parts. (a) The nucleate of the {10-12} extension twins (P1–P2), (b) the growth of {10-12} extension twins (P2–P4), and (c) the deformation band formed (P4–P5). (d) Fracture (P6).

In the first part (P1–P2), there is an elasto-plastic transition at this stage and the slip is massively activated makes the decrease in strain hardening rate slow down [43,44,45]. In the second part (P2–P4), at P2, the twins grew up in many grains, as shown in Figure 9. The twins divide the matrix into many small pieces, which brings the grain refinement effect with obvious strain hardening. Figure 6 shows that, with the increase in the strain, some grains were merged by the twinning.

**Figure 16:**
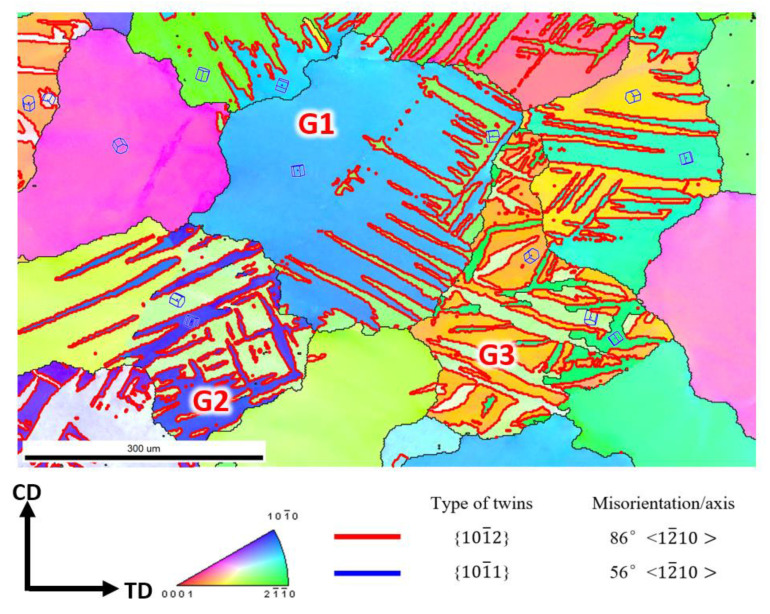
The IPF + GB map of the P3 specimen. G1 is the grain the matrix was almost covered by the twin in blue. G2 is the grain which matrix is yellow-green and twinning is blue. G3 is the matrix in yellow with two twin variants.

In Figure 16, Grain G3 has two different varieties of cross structure, which can lead to obvious strain hardening. Moreover, the different twin varieties cannot merge with each other, and their interaction will lead to more strain hardening [46]. In some grains, the twins grow fast, which have only one variety that will cover the matrix, as shown in Grain G1 in Figure 16. For Grain G1, we can clearly see the 86.5° {10-12} <11–20> TB between the matrix and the twin. Given the stress loading direction and the orientation of the matrix, we can see that the green area is the rest of the matrix, and the blue area is the twin, which means that the twinning is covering almost the whole grain, and the Grain G2 shows the same status.

In the third part, the generation of extension twins leads to a significant basal texture in the ZK60 Mg alloy, which promote the activation of pyramid slip. The massive activation of pyramid slip provided enough strain hardening and make the strain hardening rate keep a stable value, and the accumulation of slip leads to the creation of deformation bands which was shown in Figure 9d.

**Figure 17:**
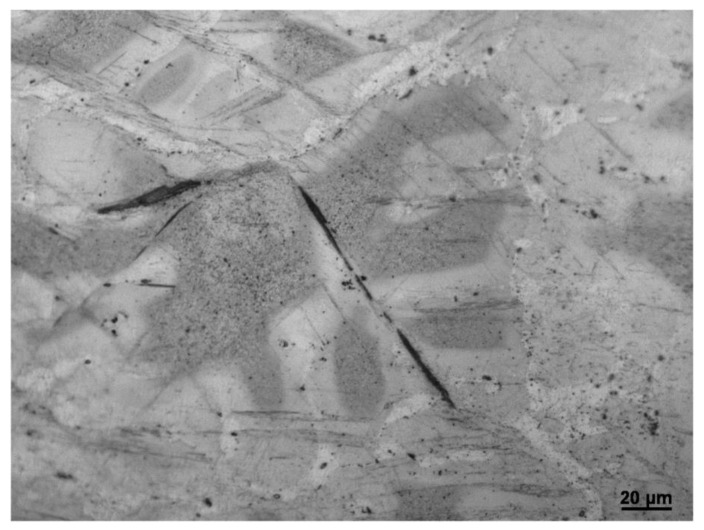
The crack in the P6 specimen.

In the fourth part, at P6, crakes occurred. In Figure 17, we can observe the crakes, and the contraction TBs usually were considered as the cracker source [46].

### 4.2. The Mechanical Property Related to Twinning

**Figure 18:**
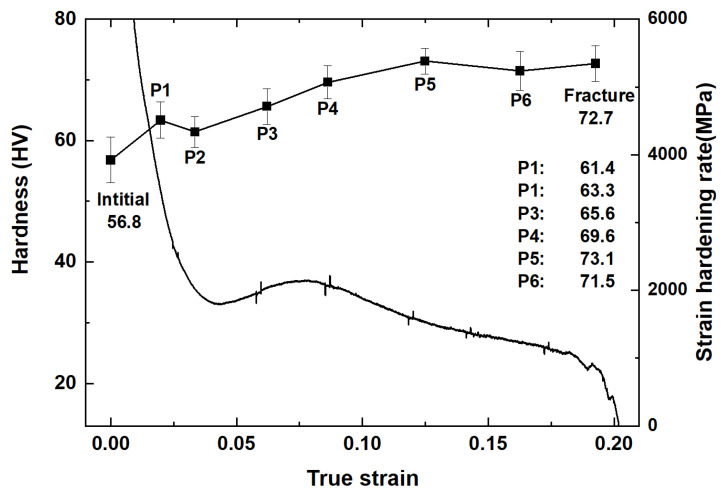
The hardness and strain hardening rate evolution with true strain.

Figure 18 shows a significant correlation between strain hardening rate and hardness. With the strain hardening rate decreasing rapidly, P1 shows that the hardness increased, then the hardness dropped a little; after P2, with the increase in the strain, the hardness continued to increase. In short, after compression, the hardness of the specimen continuously increases in comparison with the initial state.

The hardness evolution shows a strong relationship with the strain hardening rate. From Figure 6 and Figure 18, we can clearly see that the in the area around P1, the hardness of the specimen increased rapidly, which means that the dislocation activation and the interaction between twinning and dislocation bring the obvious strain hardening. In this part, the stress was quite low (~50 MPa), so the slip activated should be the basal slip.

Then, from P2–P4, the massive nucleation of the twins and the length of TB increased rapidly, dividing the matrix and the TB-dislocation interaction, and the twin-twin interaction caused the hardness to increase rapidly. In P4–P5, the twins expand to the whole matrix, which means TB as the barrier potential of dislocation activation is lost, but in this section, the activation of the basal slip and pyramid slip makes the dislocations have a strong interaction, and also makes the alloy still have the high hardness.

## 5. Conclusions

Twinning is one of the main deformation mechanisms of cast ZK60 Mg alloy during uniaxial compression at room temperature. It plays an important role in the evolution of microstructure, texture, and mechanical property. Some detailed conclusions can be draw as follows:

(1) At the beginning of deformation, the {10-12} extension twinning is one of the main deformation mechanisms and is responsible for the basal texture. Accompanied by the formation of strong basal texture in the middle and late stage of deformation, compression twinning and deformation band occurred, which is the main source of crack initiation leading to failure.
(2) Slip is also an indispensable deformation mechanism. Basal slip still kept sufficient to activate during the whole compression process. With the strain increase and texture evolution, non-basal slip gradually turned from prismatic slip to pyramidal <**c + a**> slip.
(3) During compression, the interaction between the twins, and the interaction between twin boundaries and dislocations can significantly enhance the strain hardening rate of Mg alloy, and these two interactions together with the segmentation of grains by twin boundaries improve the mechanical properties of ZK60 alloy.
(4) The fine and dense precipitates of Mg-Zn phase in ZK60 Mg alloy will not be cut when sheared by the twin boundary, but is rotated by a small angle. The precipitation may hinder the growth of twins, but promote the nucleation of twinning.

## Figures and Tables

**Table 1:** The chemical composition of ZK60 Mg alloy.

	Mg	Zn	Zr	Y
wt%	Bal.	5.56	0.58	<0.01

**Table 2:** The six compression strain points for further microstructure characterization.

	P1	P2	P3	P4	P5	P6
True Strain	0.0025	0.0275	0.055	0.091	0.136	0.187
Engineering Strain	0.0025	0.0279	0.057	0.094	0.146	0.206

**Table 3:** The fraction of LAGBs and 86.5° twin boundaries.

	P1	P2	P3	P4	P5	P6
LAGBs	0.053	0.032	0.569	0.308	0.332	0.479
86.5° ± 5°	0.170	0.576	0.676	0.414	0.181	0.154

## Data Availability

The data presented in this study are available upon request from the corresponding author. The data are not publicly available due to the requirements of related projects.
